# Non-ossifying Fibroma of Mandible in a Four-Year-Old Girl: A Case Report

**DOI:** 10.7759/cureus.36470

**Published:** 2023-03-21

**Authors:** Sandeep Khandaitkar, Gagandeep Lamba, Vrinda Kolte, Ramakrishna Shenoi, Deepankar Shukla

**Affiliations:** 1 Oral and Maxillofacial Surgery, Vidya Shikshan Prasark Mandal (VSPM) Dental College & Research Centre, Nagpur, IND; 2 Pediatric & Preventive Dentistry, Vidya Shikshan Prasark Mandal (VSPM) Dental College & Research Centre, Nagpur, IND; 3 Oral & Maxillofacial Surgery, Vidya Shikshan Prasark Mandal (VSPM) Dental College & Research Centre, Nagpur, IND; 4 Oral and Maxillofacial Surgery, Sharad Pawar Dental College and Hospital, Datta Meghe Institute of Higher Education and Research, Wardha, IND

**Keywords:** malignant fibrous histocytoma, metaphyseal fibrous defect, histiocytic xanthogranuloma, fibrous cortical defect, non-ossifying fibroma

## Abstract

Non-ossifying fibroma (NOF) is not prevelant in the mandible. It appears mostly in the long tubular bones in children and adolescents. We are presenting a case of a four-year-old girl reported to the maxillofacial department with painless swelling over the lower right side of the jaw. On the orthopantomogram (OPG), a well-defined multilocular radiolucency with a sclerotic margin was present. On computed tomography (CT), an expansile lytic lesion with cortical thinning without a breach in cortical continuity was noted. By correlating clinical and radiological features, a diagnosis of odontogenic and/or osteogenic lesion was made. The patient was considered for an excisional biopsy with curettage. On histopathology, NOF was confirmed. On postoperative follow-up, there was no sign of recurrence, and bone regeneration was significant.

## Introduction

Non-ossifying fibroma (NOF) is a benign lesion of the skeletal system that occurs mostly in long tubular bones, rarely in the mandible [1]. The distal femoral metaphysis is the most often affected minor cortical defect in children, according to radiography findings reported in the orthopaedic literature [2,3]. Jaw lesions have a female predilection, while long bone lesions have a male predilection. During the skeletal examination of healthy children, NOF was noticed on the medial aspect of one or more lower femoral metaphyses [4,5]. The NOF is categorised by the World Health Organization (WHO) as a tumor-like lesion group based on the histologic categorization of bone tumours [6,7]. Numerous descriptive synonyms based on histological origin, such as fibrous cortical defect (FCD), histiocytic xanthogranuloma, and metaphyseal fibrous defect have been used for NOF. The most prevalent benign skeletal system lesions are the FCD and the NOF. Children and teenagers are more likely to develop them. A mandibular lesion was given the name "non-ossifying fibroma" for the first time in 1979 [8]. We discussed the thorough management of a rare case of a four-year-old toddler with NOF that was diagnosed in the right mandibular angle region.

## Case presentation

**Figure 1:**
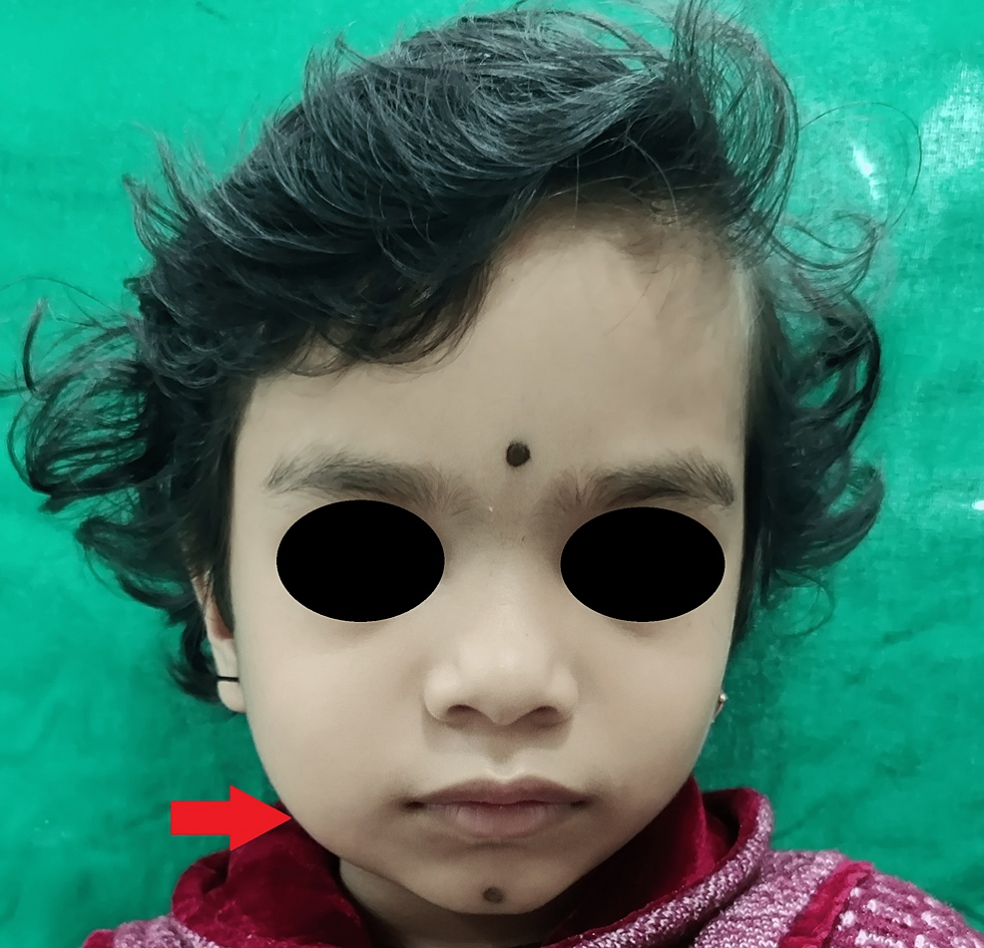
Frontal facial profile Frontal facial profile showing facial asymmetry due to the presence of diffuse swelling over the right angle region of the jaw.

A four-year-old Caucasian girl was referred to the maxillofacial surgery unit for assessment of an asymptomatic swelling over the right angle of the jaw (Figure 1).

**Figure 2:**
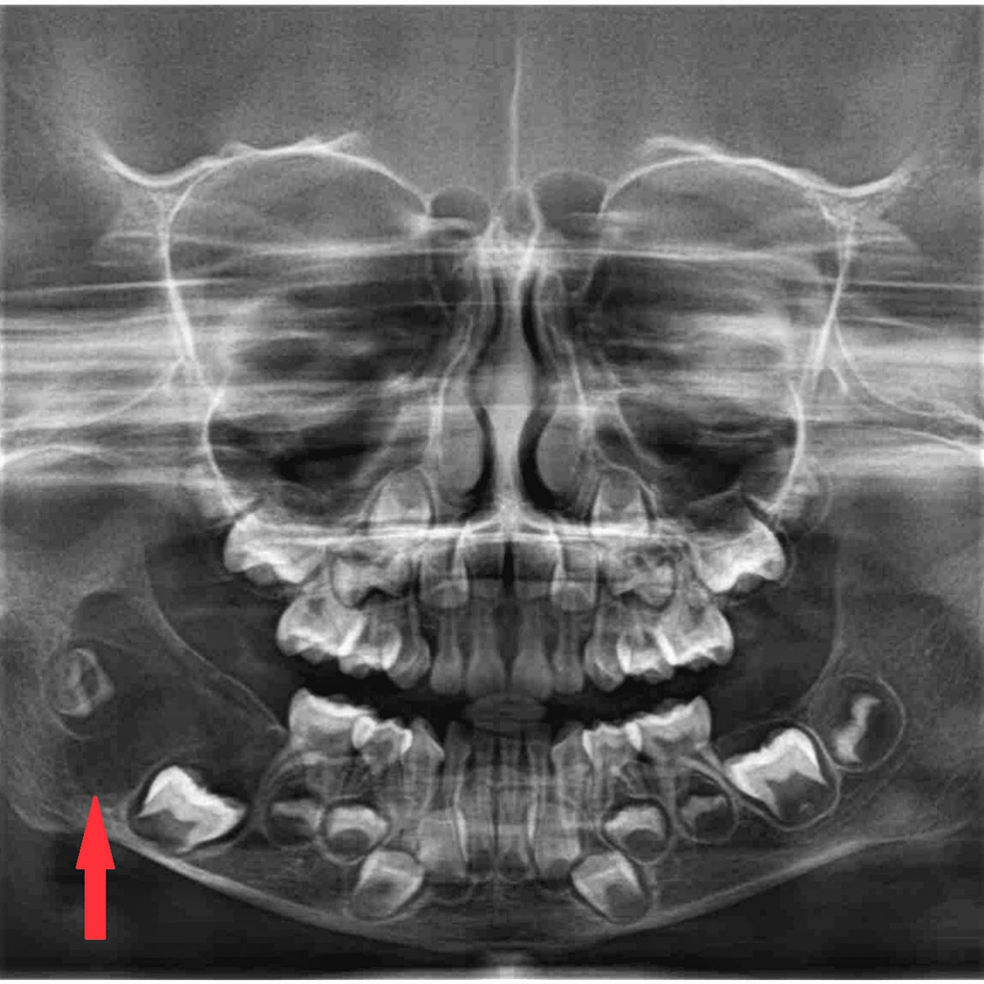
Orthopantomogram Orthopantomogram revealed a  well-defined multilocular radiolucency located at the angle region of the right mandible.

The non-tender swelling gradually increased to the current dimension within a time span of one year. There was no history of trauma due to a fall or any sports injury to the area of the chief complaint. There was no pain or trismus, and there was no significant previous medical history. There was a hard, non-tender mass palpable near the right mandibular angle. Upon intraoral examination, the overlying mucosa appeared normal in colour and texture. Cranial nerve functions were reported to be intact. There was an absence of local lymphadenopathy. A positive result from a test of pulp vitality was obtained. Orthopantomogram (Opg) revealed a well-defined multilocular radiolucency located at the angle region of the right mandible (Figure 2).

**Figure 3:**
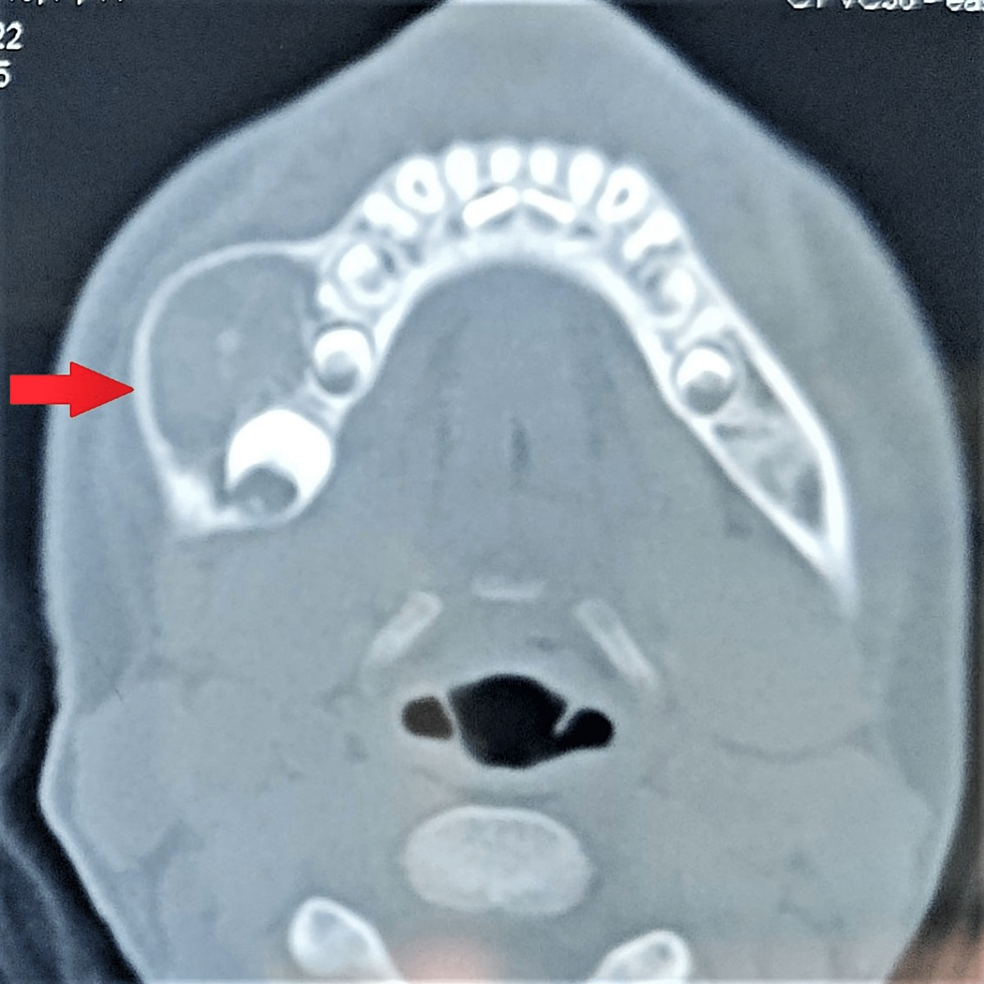
CT face plain axial section On CT face plain axial section bone window, a well-defined expansile lytic lesion was noted arising from the angle of the mandible on the right side, extending anteriorly till the deciduous molar tooth. Significant cortical thinning along with buccal expansion was noted at the perilesional area. No evidence of an obvious cortical breach was noted.

**Figure 4:**
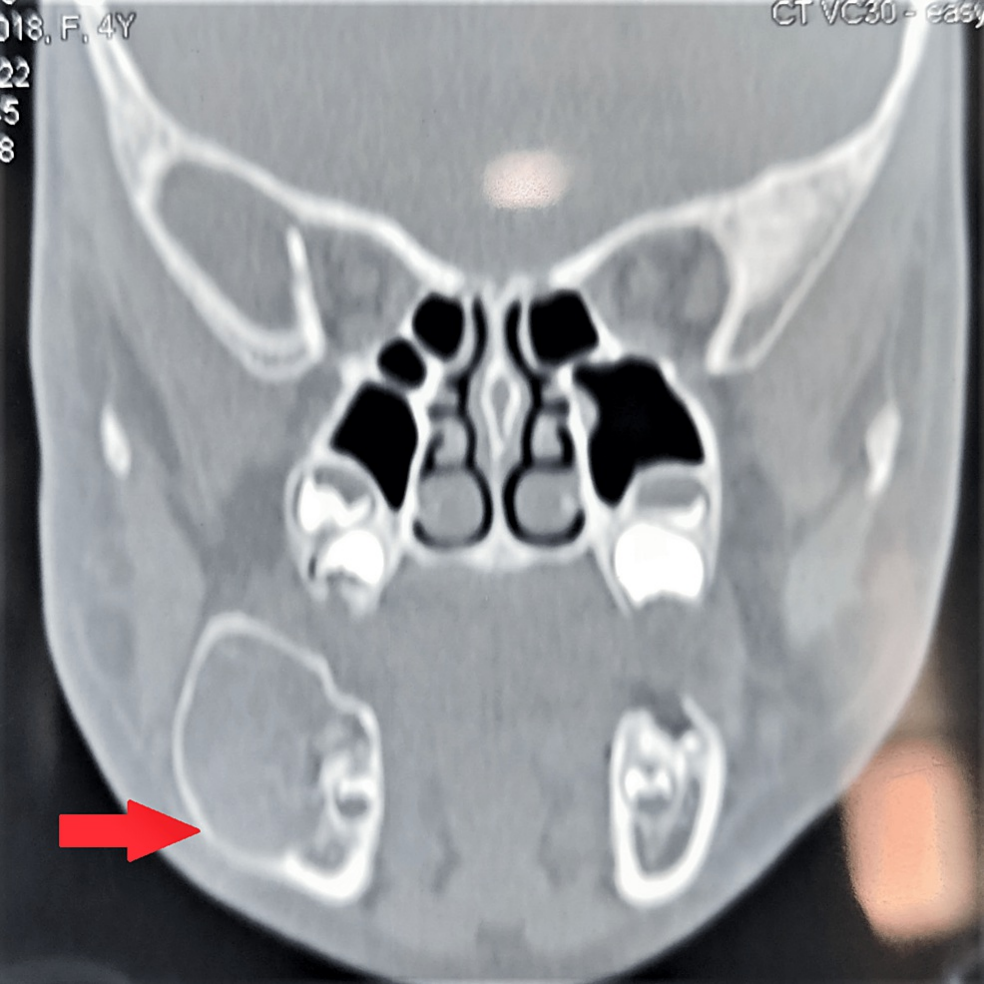
CT face plain coronal section On CT Face plain coronal section bone window, a well-defined expansile lytic lesion was noted arising from the angle of the mandible on the right side extending superiorly till the upper border of the mandible, inferior border of mandible inferiorly. Significant cortical thinning was noted at the perilesional area. No evidence of an obvious cortical breach was noted.

A well-defined expansile lytic lesion arising from the angle of the mandible on the right side, extending anteriorly till the deciduous molar tooth, superiorly till the upper border of the mandible, and inferiorly till the inferior border of the mandible and also marked buccal expansion was noted on the CT Face plain axial and coronal section bone window. Significant cortical thinning was noted at the perilesional area. No evidence of an obvious cortical breach was noted (Figures 3, 4).

A differential diagnosis was made of odontogenic and/or osteogenic lesions for dentigerous cyst, ameloblastoma based on clinical and radiological features. The alkaline phosphatase level was minimally raised. The lesion was curetted using an intraoral wards II incision under general anaesthesia, and a peripheral ostectomy was carried out.

**Figure 5:**
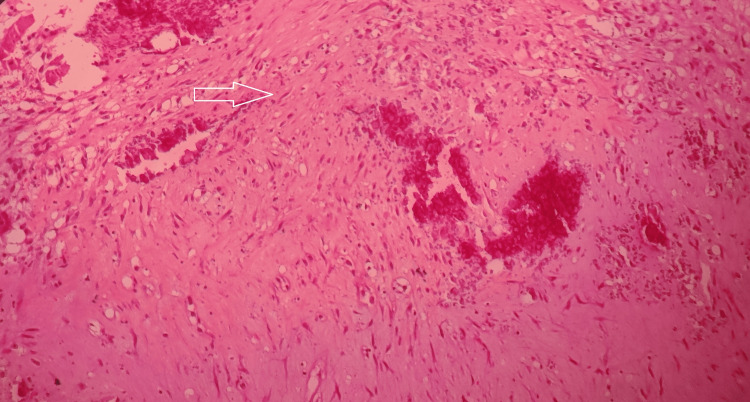
Histopathology study A stellate to spindle fibroblastic cells amidst myxoid background and reactive bone formation with osteoblastic rimming are seen. The spindle cells had sparse eosinophilic cytoplasm and oval nuclei. There was a focal cluster of osteoclast giant cells seen around the bone as well as singly scattered amidst the stroma.

Postoperatively, the formalin-fixed, paraffin-embedded tissues underwent microscopic inspection, which revealed stellate to spindle fibroblastic cells amidst a myxoid background and reactive bone formation with osteoblastic rimming. There was a focal cluster of osteoclast giant cells seen around the bone as well as scattered singly amidst the stroma. The spindle cells had sparse eosinophilic cytoplasm and oval nuclei. There were no regions of necrosis, mitoses, or unusual nuclei found (Figure 5).

**Figure 6:**
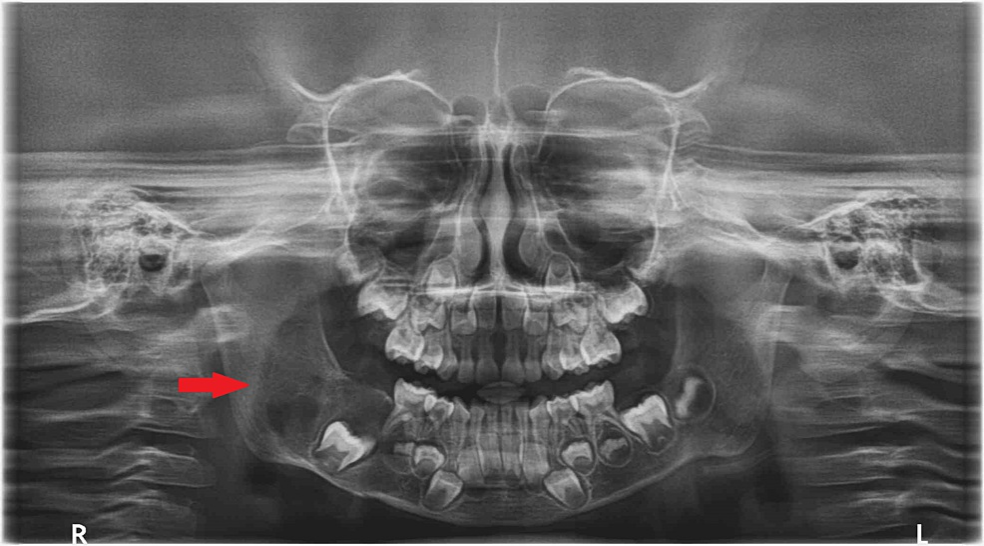
A panoramic radiograph taken during the one-year follow-up The red arrow shows persistent bone consolidation around the right mandibular angle.

There were no signs of bone production or hemosiderin pigment. The histopathologic findings and their correlation with the radiographic presentation led to the diagnosis of NOF. The patient was evaluated one month, six months, and a year after the surgery. The most recent data available, a panoramic radiograph taken during the one-year follow-up, showed persistent bone consolidation around the right mandibular angle (Figure 6). On postoperative follow-ups, the patient was asymptomatic.

## Discussion

Non-ossifying fibroma is a non-neoplastic lesion of the long tubular bone [1]. It is present eccentrically in the long bones [8]. Long tubular bone NOF has a larger diameter than gnathic NOF [9]. Long bone NOF has a male predilection, whereas as gnathic NOF has a female predilection [5]. In 2012, Bowers et al. mentioned a 1:2.3 male-to-female predilection in their review of mandibular NOF cases [1]. In 2001, Bailey et al. proposed a link between the presence of red marrow in long bone and NOF. In Gnathic NOF, the ramus and condyle are the predominant sites involved, whereas the condyle grows by an appositional bone growth [10]. In NOF, both clinical and radiological aspects can lead to a provisional diagnosis of odontogenic and/or osteogenic lesion, such as an asymptomatic, non-tender mass, a uniform expansile lesion, or the presence of cortical thinning without cortical violation and a diagnosis of NOF is made on the basis of histopathology. Whereas ameloblastoma has a soap bubble appearance, the locules present are usually larger. In the differential diagnosis of NOF, the FCD is the main entity recognized histologically. It typically presents as an osteolytic defect that is eccentrically placed.

Due to their modest size, FCDs typically have no symptoms and are subject to spontaneous regression. The FCD expands and extends into the medullary cavity by gradually separating from the growth plate, which distinctly differentiates it from NOF. NOF is larger morphologically than FCD [8]. Although NOF is mostly asymptomatic, those that are especially large may result in long-term discomfort or even a pathologic fracture.

Histologically, the presence of giant cells can lead to the differential diagnosis of benign fibrous histocytoma (BFH) and malignant fibrous histocytoma (MFH), as well as central giant cell granuloma (CGCG). BFH is usually symptomatic and affects people in their 60s and older. MFH radiologically presents as a lytic lesion with a corticol breach. MFH histologically has nuclear pleomorphism and an increase in mitotic activity. CGCG has hemosiderin deposition, erythrocyte extravasation. The giant cell arrangement is diffusely arranged in CGCG [1].

Jaw lesions of the NOF can either show enlargement or be asymptomatic. A well-demarcated multilocular radiolucency of this rare case report with sclerotic boundaries, is comparable to the normal radiographic appearance in the long bones. It is similar to previous cases reported in the literature. The isolated gnathic NOF appears to present at a later average age at diagnosis than the long bones, which may explain this difference in the age of presentation. In 2012, Bowers et al. treated a case of NOF in a 22-year-old female with curettage and peripheral ostectomy [1]. In 2011, Chrcanovic et al. managed a case of NOF in a 15-year-old male with simple curettage [8]. Large lesions may require segmental resection [10].

## Conclusions

This case report reveals an extremely rare form of NOF in a four-year-old girl who presented with painless swelling in the lower right angle region for one year. The present case highlights the significance of considering the asymptomatic nature, radiographic presentation, histopathology features, and lack of cortical breach as important factors in the diagnosis of multilocular radiolucency in the mandible. This case report adds to the growing knowledge of co-relating histopathological characteristics with clinical and radiological features. In addition, the current case describes the importance of considering curettage as a treatment modality in the management of NOF.

## References

[1] Bowers LM, Cohen DM, Bhattacharyya I, Pettigrew JC Jr, Stavropoulos MF (2013). The non-ossifying fibroma: a case report and review of the literature. Head Neck Pathol.

[2] CA J (1955). On fibrous defects in cortical walls of growing tubular bones: their radiologic appearance, structure, prevalence, natural course, and diagnostic significance. Adv Pediatr.

[3] Park JK, Levy BA, Hanley JB Jr (1982). Non-ossifying fibroma of the mandible: report of a case. J Baltimore Coll Dent Surg.

[4] Emori M, Tsuchie H, Teramoto A (2022). Non-ossifying fibromas and fibrous cortical defects around the knee - an epidemiologic survey in a Japanese pediatric population. BMC Musculoskelet Disord.

[5] Jaffe HL, Lichtenstein L (1942). Non-osteogenic fibroma of bone. Am J Pathol.

[6] Fletcher CD (2006). The evolving classification of soft tissue tumours: an update based on the new WHO classification. Histopathology.

[7] Blau RA, Zwick DL, Westphal RA (1988). Multiple non-ossifying fibromas. A case report. J Bone Joint Surg Am.

[8] Chrcanovic BR, Albanese AL, Freire-Maia B, Nunes FC, Souza PE, Gomez RS (2011). Non-ossifying fibroma (metaphyseal fibrous defect) of the mandible. Oral Maxillofac Surg.

[9] Rogozhin DV, Konovalov DM, Kozlov AS, Talalaev AG, Ektova AP (2016). Non-ossifying fibroma (metaphyseal fibrous defect) (Article in Russian). Arkh Patol.

[10] Bailey JS, Nikitakis NG, Lopes M, Ord RA (2001). Nonossifying fibroma of the mandible in a 6-year-old girl: a case report and review of the literature. J Oral Maxillofac Surg.

